# Prospective Comparison of Hypofractionated Versus Normofractionated Intensity-Modulated Radiotherapy in Breast Cancer: Late Toxicity Results of the Non-Inferiority KOSIMA Trial (ARO2010-3)

**DOI:** 10.3389/fonc.2022.824891

**Published:** 2022-05-05

**Authors:** Gustavo R. Sarria, Grit Welzel, Martin Polednik, Frederik Wenz, Yasser Abo-Madyan

**Affiliations:** ^1^ Department of Radiation Oncology, University Medical Center Mannheim, Medical Faculty Mannheim, Heidelberg University, Mannheim, Germany; ^2^ Department of Radiation Oncology, University Medical Hospital Bonn, University of Bonn, Bonn, Germany; ^3^ University Medical Center Freiburg, Medical Faculty Freiburg, Freiburg University, Freiburg, Germany

**Keywords:** breast cancer, breast-conserving surgery, whole-breast IMRT, hypofractionation, normofractionation, toxicity

## Abstract

**Purpose:**

To compare the late toxicity profile of hypofractionation and normofractionation for whole-breast radiotherapy in breast cancer (BC) patients after conserving surgery.

**Methods:**

Sixty-year-old or older patients with pTis-pT3, pN0-pN1a, M0 BC were recruited and stratified to hypofractionated (arm R-HF) or normofractionated (arm L-NF) intensity-modulated radiotherapy (IMRT), for right- and left-sided BC, respectively, in this single-center, non-randomized, non-inferiority trial. A boost was allowed if indicated. The primary outcome was the cumulative percentage of patients developing grade III fibrosis, grade I telangiectasia, and/or grade II hyperpigmentation after 2 years, with a pre-specified non-inferiority margin of 15% increase from an expected 2-year toxicity rate of 20%.

**Results:**

The Median follow-up was 4.93 (0.57–8.65) years for R-HF and 5.02 (0.65–8.72) years for L-NF (p=0.236). The median age was 68 (60–83 and 60–80) years, respectively. In total, 226 patients were recruited (107 for R-HF and 119 for L-NF), with 100 and 117 patients suitable for assessment, respectively. A boost was delivered in 51% and 53% of each arm, respectively. Median PTV volumes were 1013.6 (273–2805) cm^3^ (R-HF) and 1058.28 (315–2709) cm^3^ (L-NF, p=0.591). The 2-year primary endpoint rate was 6.1% (95% CI 1.3-11.7, n=5 of 82) and 13.3% (95% CI 7-20.2, n=14 of 105), respectively (absolute difference -7.2%, one-sided 95% CI ∞ to -0.26, favoring R-HF). No local recurrence-free- or overall-survival differences were found.

**Conclusion:**

In this prospective non-randomized study, hypofractionation did not have higher toxicity than normofractionated whole-breast IMRT.

## Highlights

• Toxicity after hypofractionated whole-breast radiotherapy is not inferior to normofractionation
• Hypofractionation yields approximately -7% overall less undesired toxic events
• Toxicity results are favorable despite employing boost volumes
• No significant difference in local control or survival was found

## Introduction

Breast cancer (BC) is the most common malignant disease in the female population with a share of 28.2% (523,000) of all newly diagnosed cases, and about 16.2% (138,000) of affected patients in Europe will die as a consequence of the disease. Moreover, the incidence and mortality per 100,000 inhabitants are 100.9 and 21.8, respectively (1).

In Germany, about 71,888 (25.9%) new breast cancer cases occur annually; therefore, BC represents the most common malignancy in German women. Each year, about 19,376 of these patients die because of BC, accounting for 7.8% of the total cancer-related deaths. In women aged 35 to 55 years, breast cancer is the most common cause of death. The overall 5-year survival rate is estimated at about 77% (2, 3).

Since the early 1990s, breast-conserving therapy (BCT) has become an accepted treatment option for stage I and II BC patients (4). Multiple retrospective and prospective randomized studies have examined the long-term equivalency of BCT compared to mastectomy regarding disease-free and overall survival (5–8). The need for adjuvant breast irradiation as part of BCT was repeatedly proven in the treatment of early-stage disease. The updated NSABP-B06-study data showed a 39.2% local recurrence rate without radiation therapy versus 14.3% with adjuvant radiotherapy (5). Furthermore, more recently published data support these findings showing further improvements in treatment outcomes (9).

A meta-analysis of the Early Breast Cancer Trialists’ Collaborative Group (EBCTCG) in 2000 (6) and 2005 (7) showed reduced mortality and increased overall survival for patients treated with adjuvant breast irradiation.

The main advantages of BCT are better cosmetic, lower toxicity results, and reduced psychological and emotional load when compared to radical surgical approaches. This premise has been confirmed by the reported satisfaction levels amongst patients treated with conservative surgery plus radiotherapy, in favor of this method (10). However, classical long radiotherapy times, taking about 5-7 weeks, could also be considered as inconvenient for many patients. A shorter scheme would be beneficial not only for the patient but also for radiotherapy facilities. Patient convenience with reduced overall hospital visits is mainly in the form of reduced travel costs and psychological welfare due to shorter treatment time and faster return to regular lifestyle (11, 12). Convenience for centers comes from reduced workload, costs, and eventually optimized utilization of available resources (13, 14).

The proven effect of whole-breast hypofractionation (HF) in this setting of patients (15–17) has raised a question in terms of cosmesis and toxicity differences compared to standard schemes (18). Recent published prospective data have shown a benefit in terms of acute toxicity and quality of life (QOL) profiles, with 6- and 8-week overall improvement (19, 20). These publications support previously released data that demonstrated better 6-month physical and social outcomes in favor of HF against normofractionation (NF) (21). A growing body of evidence in this regard has been published in the past decade (15).

Herein, we report the acute and late toxicity outcomes of a prospective trial, studying patients treated with tangential IMRT (tIMRT) fields in normo- and hypofractionation schemes, addressing the hypothesis of non-inferiority for the latter.

## Methods

### Patient Selection and Procedures

This was a prospective, single-center, open-label, non-randomized, two-arm study comparing normofractionated and hypofractionated radiotherapy in patients with breast cancer using tangential IMRT techniques. Inclusion criteria encompassed unilateral breast-cancer 60-years-old or older patients with disease stages pTis-pT3, pN0-pN1a, M0 (TNM 7^th^ edition) after breast-conserving surgery, regardless of the molecular profile. Patients were allocated to receive hypofractionation (arm R-HF, 40.05/2.67Gy in 15 fractions) for right-sided primaries and normofractionation (arm L-NF, 50/2Gy in 25 fractions) for left-sided primaries. In both arms, only patients between 60 and 69 years were to receive a boost (16/2Gy in 8 fractions), if indicated. No axillary irradiation was considered, according to the German guidelines at the time point the trial was initiated.

Organs at risk and target volumes were contoured according to the RTOG guidelines. In both groups, the tangential intensity-modulated radiotherapy (IMRT) technique, aiming to achieve optimal dose homogeneity, was applied. Follow-ups were scheduled at post-treatment first and 6^th^ weeks, 6^th^ month, 12^th^ month, and annually thereafter. A non-blinded physician-based (radiation oncologist and gynecologist) assessment was collected at every visit.

### Endpoints

The primary endpoint was late toxicity, defined as the cumulative rate of patients manifesting grade III fibrosis, grade I telangiectasia, and/or grade II hyperpigmentation 2 years after radiotherapy treatment, according to the LENT-SOMA classification, compared to baseline characteristics. This combination was selected in order to comprehensively assess the most frequently patient-reported symptoms (22) and based on institutional observational data. Secondary points of interest included rates of retraction, breast edema, ulcer, arm edema, and breast pain, classified under the same scale. Additionally, dermatitis, pneumonitis, dyspnea, and cough, classified after the CTCAE v.3.0., were evaluated until the sixth post-treatment week. The 5-year local-recurrence free survival (LRFS, defined as any in-breast recurrence) and estimated overall survival (OS) were additionally analyzed.

### Statistical Design and Analysis

A sum of grade III fibrosis, grade III telangiectasia, and grade II hyperpigmentation of approximately 20% was expected after 2 years. A protocol amendment was incorporated to assess grade I telangiectasia, instead of III, as no higher grade was observed during the follow-up period. The sample size estimated for this non-inferiority trial, 226 patients, was calculated to exclude a difference between the percentages of patients with toxicity in L-NF and R-HF arms of more than 15% (one-sided 95% confidence interval [CI] ∞ to 15%), with a power of 80%. This calculation assumed an equal accrual number in arms R-HF and L-NF and a dropout rate of 22% during the first 2 years of the study. Corresponding to the non-inferiority design, the result for the primary endpoint is presented as the difference between the percentages with a one-sided 95% CI. This 2-year toxicity rate difference was obtained by subtracting the result of R-HF arm from the L-NF arm outcomes. The 95% CI for the 2-year primary endpoint rates was calculated using the percentile method based on 10,000 bootstrap samples.

Baseline characteristics in each study group were analyzed as frequencies and percentages for categorical variables and as medians and ranges for continuous variables, as appropriate. A cross-sectional analysis was used for rates and frequencies of all adverse events and their corresponding grading. The chi-square and Fisher’s exact tests were employed for outcome comparison, applying the Bonferroni correction when required. LRFS and OS differences between both arms were assessed through the log-rank test. A two-tailed p value of less than 0.05 was considered indicative of statistical significance. Data were analyzed with SPSS (IBM Corp. Released 2017. IBM SPSS Statistics for Windows, Version 25.0. Armonk, NY: IBM Corp.).

### Ethics Statement

The Institutional Review Board (IRB), according to local protocols, approved this work (nr. 2009-348Str.-MA) prior to initiation. Consent to participate was obtained from each patient before inclusion in this trial. This investigation was performed according to standards of the Declaration of Helsinki. Trial identifiers: NCT01403779 and ARO2010-3.

## Results

### Patient Features

Median follow-up was 4.93 (0.57–8.65) years for arm R-HF and 5.02 (0.65–8.72) years for arm L-NF (p=0.236). Median patient age was 68 (60–83 and 60–80) years, respectively. In total, 226 patients were recruited (107 for arm R-HF and 119 for arm L-NF) between July 2010 and February 2017. Nine (R-HF: 7, L-NF: 2) withdrew consent prior to treatment, yielding 100 and 117 patients suitable for analysis in each respective group. During the planned first 2 years, 22 patients (R-HF: 14, L-NF: 8) were lost to follow-up and 17 (A: 11, B: 6) withdrew consent to participate in the trial. The total lost/dropouts were 25 in arm R-HF and 14 in arm L-NF (p=0.021). Distribution according to TNM status for both arms was 12% and 8.5% for Tis, 55% and 68.4% for T1, 31% and 23.1% for T2, and 2% and 0% for T3, respectively. Lymph nodes were positive (N1a) in 8% and 7.7% for both arms. Regarding tumor bed boost, 51% and 53% received it on each arm. Chemotherapy was delivered in 17% of patients in arm R-HF and 19.6% in arm L-NF (p=0.526). Likewise, hormone treatment (HT) was administered in 79% and 87.2% (p=0.106), respectively. Median PTV total volumes were 1013.6 (273–2805) cm^3^ in arm R-HF and 1058.28 (315–2709) cm^3^ in arm L-NF (p=0.591). Further patient characteristics are displayed in **Table 1**.

**Table 1:** Baseline cohort characteristics.

Characteristics	Arm A	Arm B	p value
Recruited	107	119	0.425
Median follow-up	4.93 [0.57 - 8.65]	5.02 [0.65 - 8.72]	0.236
Age (median) Age ≥ 70	68 [60 - 83] 43%	68 [60 - 80] 40.2%	0.798 0.673
Included in the analysis	100	117	
**Currently smoking**			
Yes	13 (13%)	10 (8.5%)	0.495
No	84 (84%)	101 (86.3%)	
Unknown	3 (3%)	6 (5.1%)	
**Previously smoked**			
Yes	40 (40%)	41 (35%)	0.615
No	57 (57%)	70 (59.8%)	
Unknown	3 (3%)	6 (5.1%)	
**BMI**			
Normal	43 (43%)	54 (46.2%)	0.092
Overweight	39 (39%)	31 (26.5%)	
Obese	18 (18%)	32 (27.4%)	
**Location**			
UOQ	57 (57%)	60 (51.3%)	0.263
UIQ	16 (16%)	23 (19.7%)	
LOQ	13 (13%)	9 (7.7%)	
LIQ	8 (8%)	19 (16.2%)	
Central	6 (6%)	6 (5.1%)	
**T stage**			
Tis	12 (12%)	10 (8.5%)	0.119
T1mi	2 (2%)	0 (0%)	
T1a	6 (6%)	5 (4.3%)	
T1b	13 (13%)	25 (21.4%)	
T1c	34 (34%)	50 (42.7%)	
T2	31 (31%)	27 (23.1%)	
T3	2 (2%)	0 (0%)	
**N stage**			
N0	92 (92%)	108 (92.3%)	0.993
N1	8 (8%)	9 (7.7%)	
**Histology**			
DCIS	12 (12%)	10 (8.5%)	0.401
Invasive	88 (88%)	107 (91.5%)	
**Chemotherapy**			
No ChT	83 (83%)	94 (80.3%)	0.526
Neoadjuvant ChT	2 (2%)	6 (5.1%)	
Adjuvant ChT	15 (15%)	17 (14.5%)	
**Hormone therapy**			
No HT	21 (21%)	15 (12.8%)	0.106
HT	79 (79%)	102 (87.2%)	
**Radiotherapy**			
Boost	51 (51%)	62 (53%)	
Median time to RT in days (no ChT)	40 [24 - 126]	39 [19 - 90]	0.51
Median time to RT in days (ChT)	174 [132 - 254]	184 [63 - 253]	0.664
Median V >107% (cm3)	3.1 [0-53]	2 [0 - 40]	0.118
Median PTV volume (cm3)	1013.6 [273 - 2805]	1058 [315 - 2709]	0.591

BMI, body-mass index; UOQ, upper-outer quadrant; UIQ, upper-inner quadrant; LOQ, lower-outer quadrant; LIQ, lower-inner quadrant; DCIS, ductal carcinoma in-situ; ChT, chemotherapy; HT, hormone therapy; RT, Radiotherapy; V > 107%, target volume receiving > 107% of the prescribed dose; PTV, planning target volume.

Staging performed according to the TNM AJCC 7^th^ edition criteria.

### Toxicity and Clinical Outcomes

**Figure 1:**
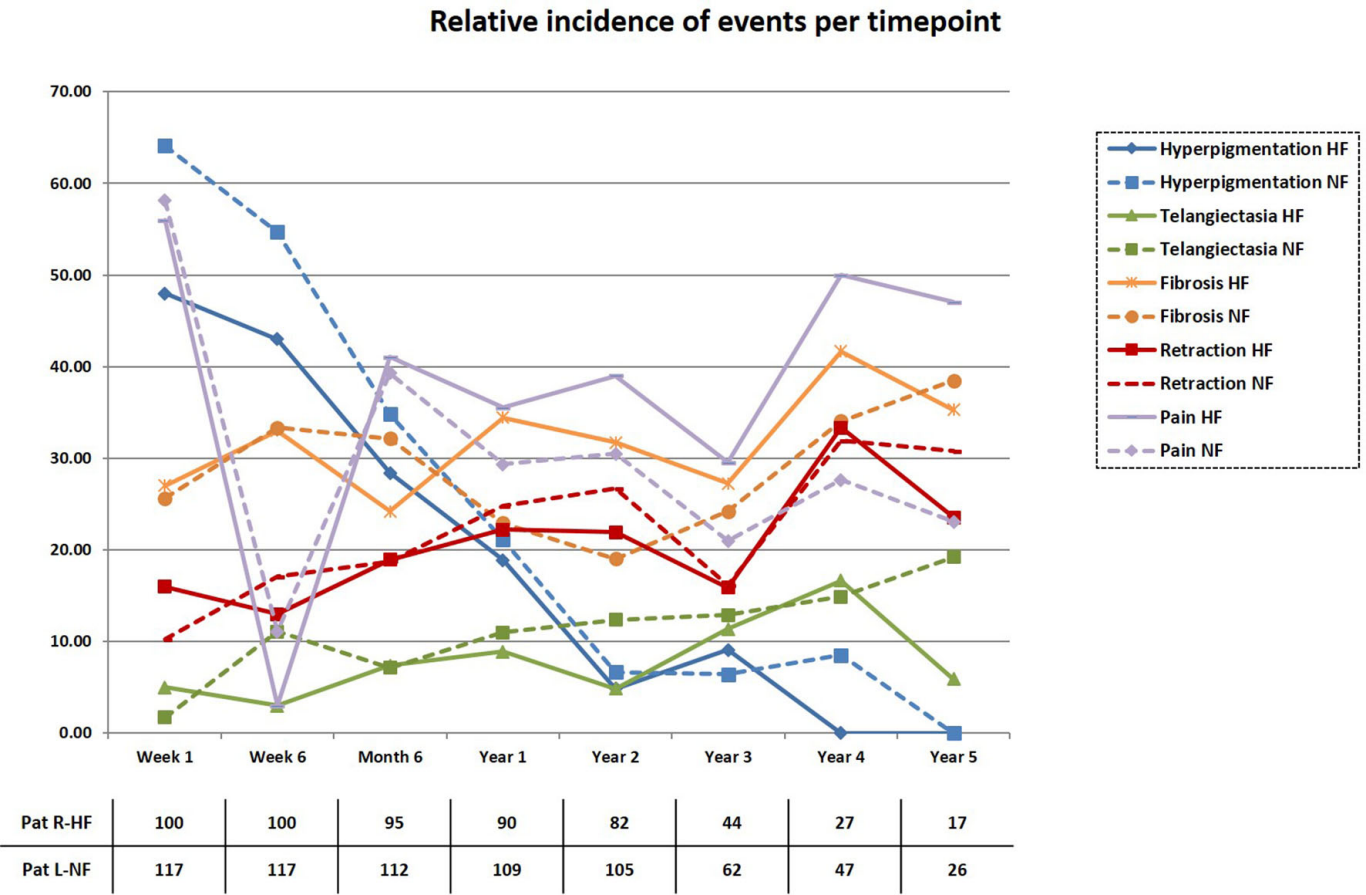
Incidence of secondary events. The cumulative incidence of patients developing secondary events (GI-III) is displayed in a cross-sectional fashion for each treatment arm and time point.

The percentage of patients with grade III fibrosis, grade I telangiectasia, and/or grade II hyperpigmentation after 2 years was 6.1% (95% CI 1.3 to 11.7, n = 5 of 82 patients) in arm R-HF and 13.3% (95% CI 7 to 20.2, n = 14 of 105 patients) in arm L-NF. The absolute difference was -7.2% (one-sided 95% CI ∞ to -0.26) favoring arm R-HF. Significant toxicity differences, according to time point and grading for both arms were 3% and 10% GII hyperpigmentation at 1^st^ week (p=0.017), 3% and 11.1% GI telangiectasia at 6^th^ week (p=0.023), 34% and 19.7% G0 dermatitis at 1^st^ week (p=0.034), and 4% and 0% GII pneumonitis at 6^th^ week (p=0.044), respectively. Additionally, grade III fibrosis and breast pain were recorded in 1.2% and 1% (p=0.077) and in 3.7% and 0% (p=0.227) at 2-year control in each arm, respectively. The overall relative incidences are shown in **Figure 1**, displayed in a cross-sectional fashion. When comparing to baseline status for each arm, significant changes were obtained at 6-month follow-up control for lymphedema (+2%, p=0.004) and 2-year control for retraction (+16%, p=0.006) in arm L-NF. No other significant changes at these time points were observed for other events. Detailed description of secondary events with statistical significance of differences between arms and further comparisons to baseline characteristics can be found in **Appendix 1**.

**Figure 2:**
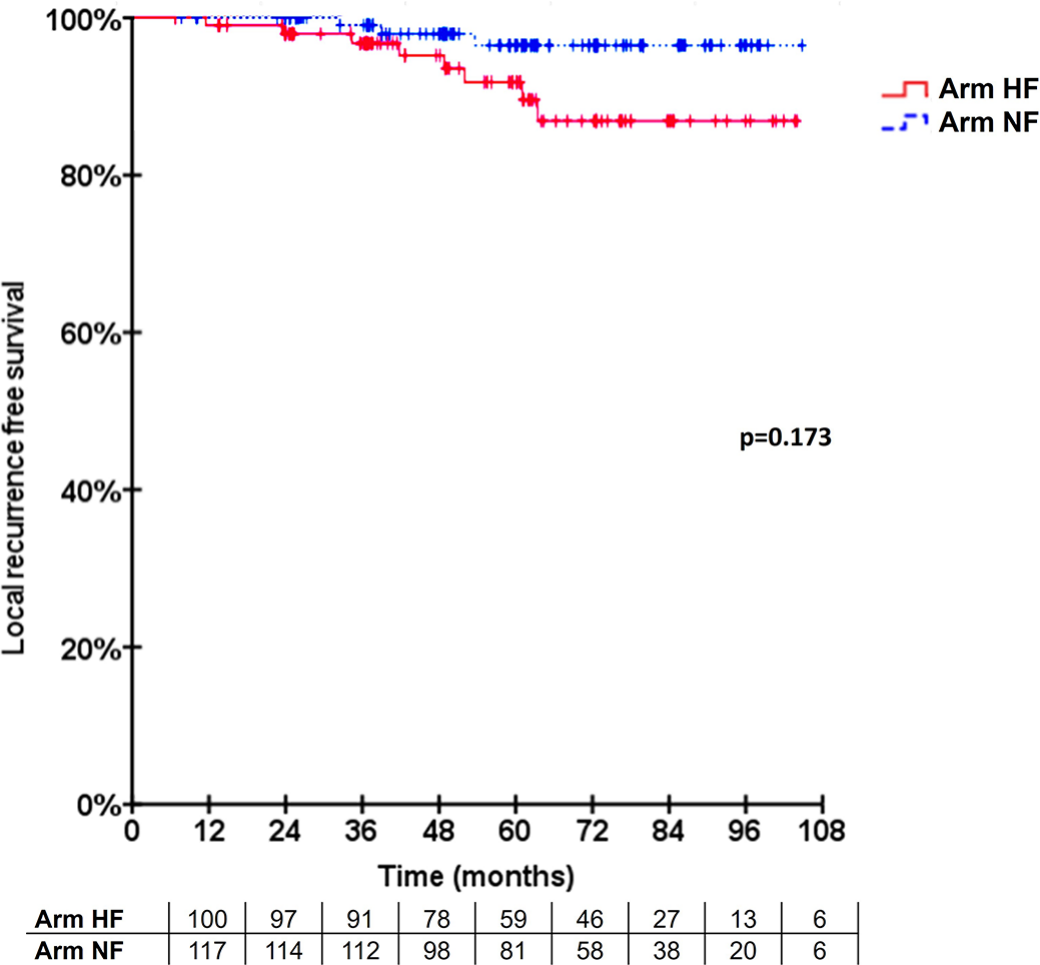
Cumulative local recurrence rate. Longitudinal local control displayed according to the Kaplan-Meier method.

**Figure 3:**
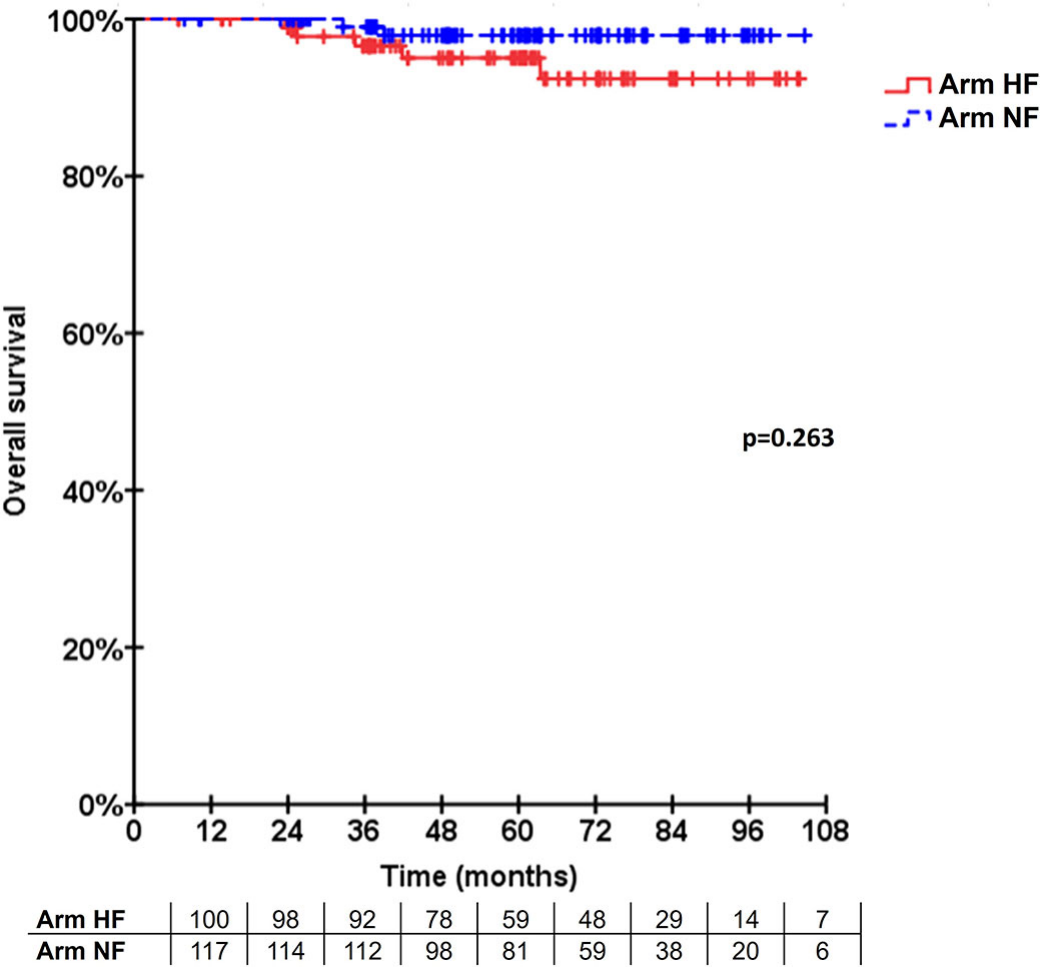
Overall survival. Kaplan-Meier curves for the estimated overall survival.

The 5-year LRFS was 91.8% and 96.5% for Arms R-HF and L-NF (n=5), respectively. No statistically significant difference was seen (p=0.173, **Figure 2**). One recurrence case in arm R-HF was documented as Paget disease, occurring after 4 years, and included in the analysis, according to protocol. In addition, two patients with locally recurrent disease were allocated in this same arm, both with triple-negative breast cancer (TNBC), while only one of them received adjuvant chemotherapy. Two hormone-sensitive BC patients who received HT failed locally, one in each treatment arm. The estimated 5-year OS was 95.0% in arm R-HF and 97.9% in arm L-NF (p=0.263, **Figure 3**).

## Discussion

This study contributes to other recently published trials exclusively designed for assessing differences, in term of late toxicity, in BC patients treated with hypo- or normofractionation. Since the publication of the START A, B, and Canadian trials, increasing interest for hypofractionation modalities in BC have greatly increased (23). Detailed data from the 10-year follow-up START and Canadian trials already highlighted the improvement of patients receiving hypofractionation in terms of breast shrinkage, breast induration, telangiectasia, breast edema, arm edema, and shoulder stiffness; however, no major details about onset times were given (15, 16).

Seeking to improve the availability and accuracy of predictive tools and in order to grant better patient-informed decisions, our group carried this investigation to provide further details on expected secondary effects when selecting hypo- or normofractionation. One of the strengths of these data lies on the detailed description of event occurring, being a powerful tool during follow-up controls. Under the period of this investigation, remarkable shifts in the breast-cancer treatment paradigm occurred. Recently published data from the FAST and FAST-Forward trials support the implementation of even shorter breast irradiation schemes with comparable oncological outcomes, albeit with a higher incidence of breast fibrosis (24, 25). Furthermore, other strategies such as intraoperative radiotherapy during breast conserving surgery, as in the TARGIT-A trial, might confer lower toxicity rates and higher patient convenience, while being less costly for healthcare systems, maintaining acceptable local control rates and reducing non-breast cancer deaths, in well-selected cases (26–29). Notwithstanding, we consider our results to be of use in cases where shifting toward these new practices could be delayed due to medical or non-medical factors (30, 31).

As per the primary outcome, the non-inferiority criterion was met. The same tendency toward an improvement in toxicity pattern was found with hypofractionation, as compared to the START and Canadian trials, although no conclusions could be drawn from it, due to the statistical design of the trial. Besides supporting these prior results, our study incorporates the development profile of other secondary events, such as hyperpigmentation, dermatitis, breast pain, and pneumonitis, which at certain time points were significantly lower in the hypofractionation arm. Noteworthy, most of these events were equal to or lower than GII toxicity and might not have a major clinical implication but could indeed affect the patients’ QOL. More recently published studies have reported similar results. The HYPO trial, a large multi-center phase III study, including patients from Denmark, Germany, and Sweden, recently reported their outcomes, in terms of toxicity and cosmesis. The primary endpoint was breast induration (surrogate of fibrosis), which, when combining both G II and III, resulted in 9% at 3 years for hypofractionation against 11.8% (32). Similar to our outcomes, the overall breast induration rate of 6.1% reported by *Wang et al.* at 5 years further supports the hypofractionation utilization (33). It must be noted that our reporting on combined fibrosis, telangiectasia, and hyperpigmentation does not ease a comparison between the abovementioned studies. Selecting a combination of events as a single endpoint obeys to a comprehensive patient-based perception of post-treatment changes. Furthermore, variable study designs impair any possible comparison. At the time point this study was designed, an expected 20% rate of combined events was decided upon institutional data from patients mostly treated during the 3D era. This factor could partially explain the lower rates herein obtained. Moreover, patients undergoing chemotherapy during the first years of the past decade might have received different combinations, currently discontinued, which may have played a role in this observation. Adding this knowledge to the available data could help in reaching a better informed decision and in understanding the onset pattern of these secondary adverse events. Similar experiences are currently being published for similar or other BC-related treatment settings (34, 35).

Although the LRFS and OS are none-planed endpoints to this trial, they are still to be highlighted. The observed differences in failures between both arms were not statistically significant. Despite the slight tendency against hypofractionation (n=4 vs n=1), it should be remarked that one patient in this arm developed Paget disease; however, due to protocol considerations, was included in the LRFS analysis. Three patients in arm R-HF presented with TNBC (one of them recurred as Paget’s disease), of which only one received adjuvant chemotherapy. This leaves one patient with hormone-sensitive BC treated with HT, who failed locally, in each arm. The 5-year OS showed again a slight numeric difference in favor of arm L-NF (n=5 vs n=2). This difference could be attributed to two cases in arm R-HF, who developed secondary glioblastoma and metastatic colon carcinoma. Nevertheless, these outcomes are in line with the abovementioned trials’ results.

Inherent limitations to this study include its single-center, non-randomized nature and stratification according to localization. As this study was first conceptualized in 2009, the decision on allocating patients per laterality obeys to the then mostly-unknown long-term effects of hypofractionation on cardiac structures. Thus, a rather conservative approach was selected upon this concern. According to its initial statistical design, the study has not been powered to draw conclusions in terms of superiority of the hypofractionated versus the normofractionated arm. Additionally, these results in patients over 60 years old are not representative of younger patients, as different healing patterns and tissue regeneration capability could potentially alter the outcomes. Forbye, current trends for low-risk elderly patients (≥ 65-70 years old) include, yet controversial due to increased local failure rates, radiotherapy omission (36–38). The addition of boost in over half of the patients represents another issue of concern *per se*. As this trial was started and according to institutional standards, all patients receiving a boost irradiation volume had a 16 Gy in 2 Gy per fraction prescription, with no accepted hypofractionation boost scheme at the time, possibly altering outcomes in arm R-HF. Despite this feature, no major difference as compared to the START trials was observed, considering that 60.6% and 42.6% of patients received a tumor bed boost in both START A and B trials, respectively. Similar patterns were found in more recently published studies, specifically designed to address this hypothesis. Moreover, our findings are yet aligned with the Canadian trial, in terms of overall toxicity results. In addition, the patients recruited in this trial received current systemic therapy schemes, compared to older trials, eliminating the bias related to this factor. Despite being initially planned and powered to be a two-year analysis, ~50% of the patients still attend controls after 2 years, opening the possibility of expanding further assessments in terms of toxicity, cosmesis, and disease-control outcomes with larger follow-up periods. Furthermore, an excellent documentation standard and a relatively low rate of dropouts during the first 2 years have allowed us to report solid information on this endpoint.

Data analysis on cosmesis and QOL is currently ongoing and will be reported separately.

## Conclusion

In this prospective non-randomized study, hypofractionation did not have higher toxicity than normofractionated whole-breast IMRT.

## Data Availability Statement

The raw data supporting the conclusions of this article will be made available by the authors, without undue reservation.

## Ethics Statement

The studies involving human participants were reviewed and approved by the Institutional Review Board (IRB) - University Medical Center Mannheim, according to local protocols, approved this work (nr. 2009-348Str.-MA) prior to initiation. Written informed consent was obtained from each patient before inclusion in this trial. This investigation was performed according to standards of the Declaration of Helsinki. Trial identifiers: NCT01403779 and ARO2010-3.

## Author Contributions

GS: data collection and curation. Manuscript drafting, formatting, and editing. GW: data curation and analysis, manuscript review. MP: data collection and manuscript review. FW: study conceptualization, manuscript review. YA-M: study conceptualization, manuscript editing, and review. All authors contributed to the article and approved the submitted version.

## Conflict of Interest

GS: Personal fees and travel expenses from Carl Zeiss Meditec AG, personal fees from Roche Pharma AG, personal fees from MedWave Clinical Trials, travel costs from Guerbet SA, not related to this work. FW: Personal fees from Roche Pharma AG and Eli Lilly and Company, grants and others from Carl Zeiss Meditec AG and Elekta AB, a patent by Carl Zeiss Meditec AG, outside the submitted work.

The remaining authors declare that the research was conducted in the absence of any commercial or financial relationships that could be construed as a potential conflict of interest.

The handling editor declared a declared a past co-authorship with one of the authors FW.

## Publisher’s Note

All claims expressed in this article are solely those of the authors and do not necessarily represent those of their affiliated organizations, or those of the publisher, the editors and the reviewers. Any product that may be evaluated in this article, or claim that may be made by its manufacturer, is not guaranteed or endorsed by the publisher.
