# Supplementary material for: Prospective Comparison of Hypofractionated Versus Normofractionated Intensity-Modulated Radiotherapy in Breast Cancer: Late Toxicity Results of the Non-Inferiority KOSIMA Trial (ARO2010-3)
Source: Front Oncol. 2022 May 5;12:824891. doi: 10.3389/fonc.2022.824891 (PMC9117716; doi:10.3389/fonc.2022.824891)
Supplement: Supplementary file 1 [file DataSheet_1.pdf]

Appendix 1: Relative and absolute number of events per treatment arm and grading

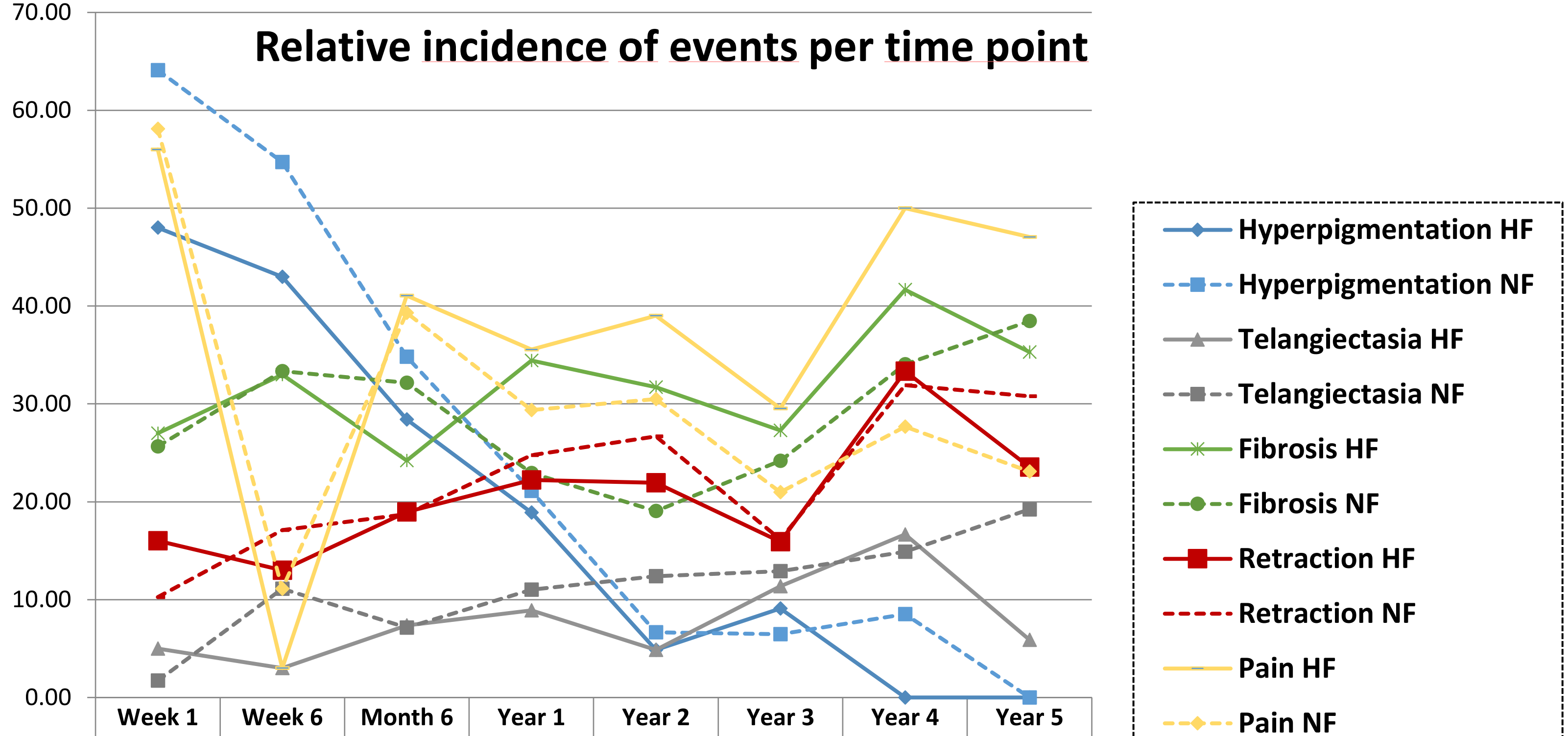

	Week 1	Week 6	Month 6	Year 1	Year 2	Year 3	Year 4	Year 5
Hyperpigmentation HF	48.0	43.0	28.4	18.9	4.9	9.1	0.0	0.0
Hyperpigmentation NF	64.1	54.7	34.8	21.1	6.7	6.5	8.5	0.0
Telangiectasia HF	5.0	3.0	7.4	8.9	4.9	11.4	16.7	5.9
Telangiectasia NF	1.7	11.1	7.1	11.0	12.4	12.9	14.9	19.2
Fibrosis HF	27.0	33.0	24.2	34.4	31.7	27.3	41.7	35.3
Fibrosis NF	25.6	33.3	32.1	22.9	19.0	24.2	34.0	38.5
Retraction HF	16.0	13.0	18.9	22.2	22.0	15.9	33.3	23.5
Retraction NF	10.3	17.1	18.8	24.8	26.7	16.1	31.9	30.8
Pain HF	56.0	3.0	41.1	35.6	39.0	29.5	50.0	47.1
Pain NF	58.1	11.1	39.3	29.4	30.5	21.0	27.7	23.1

Absolute events (n) per time point

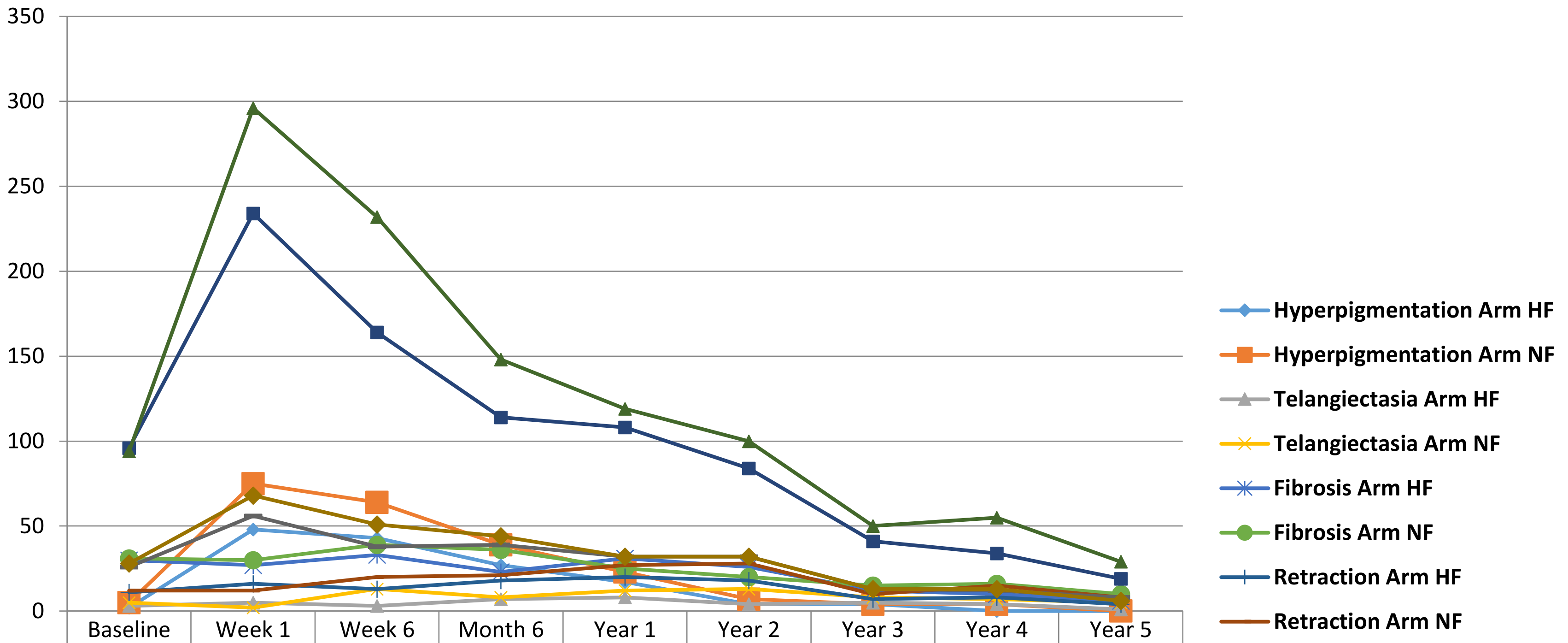

	Baseline	Week 1	Week 6	Month 6	Year 1	Year 2	Year 3	Year 4	Year 5
Hyperpigmentation Arm HF	2	48	43	27	17	4	4	0	0
Hyperpigmentation Arm NF	5	75	64	39	23	7	4	4	0
Telangiectasia Arm HF	3	5	3	7	8	4	5	4	1
Telangiectasia Arm NF	5	2	13	8	12	13	8	7	5
Fibrosis Arm HF	30	27	33	23	31	26	12	10	6
Fibrosis Arm NF	31	30	39	36	25	20	15	16	10
Retraction Arm HF	11	16	13	18	20	18	7	8	4
Retraction Arm NF	12	12	20	21	27	28	10	15	8
Pain Arm HF	26	56	38	39	32	32	13	12	8
Pain Arm NF	28	68	51	44	32	32	13	13	6
Total HF	96	234	164	114	108	84	41	34	19
Total NF	94	296	232	148	119	100	50	55	29

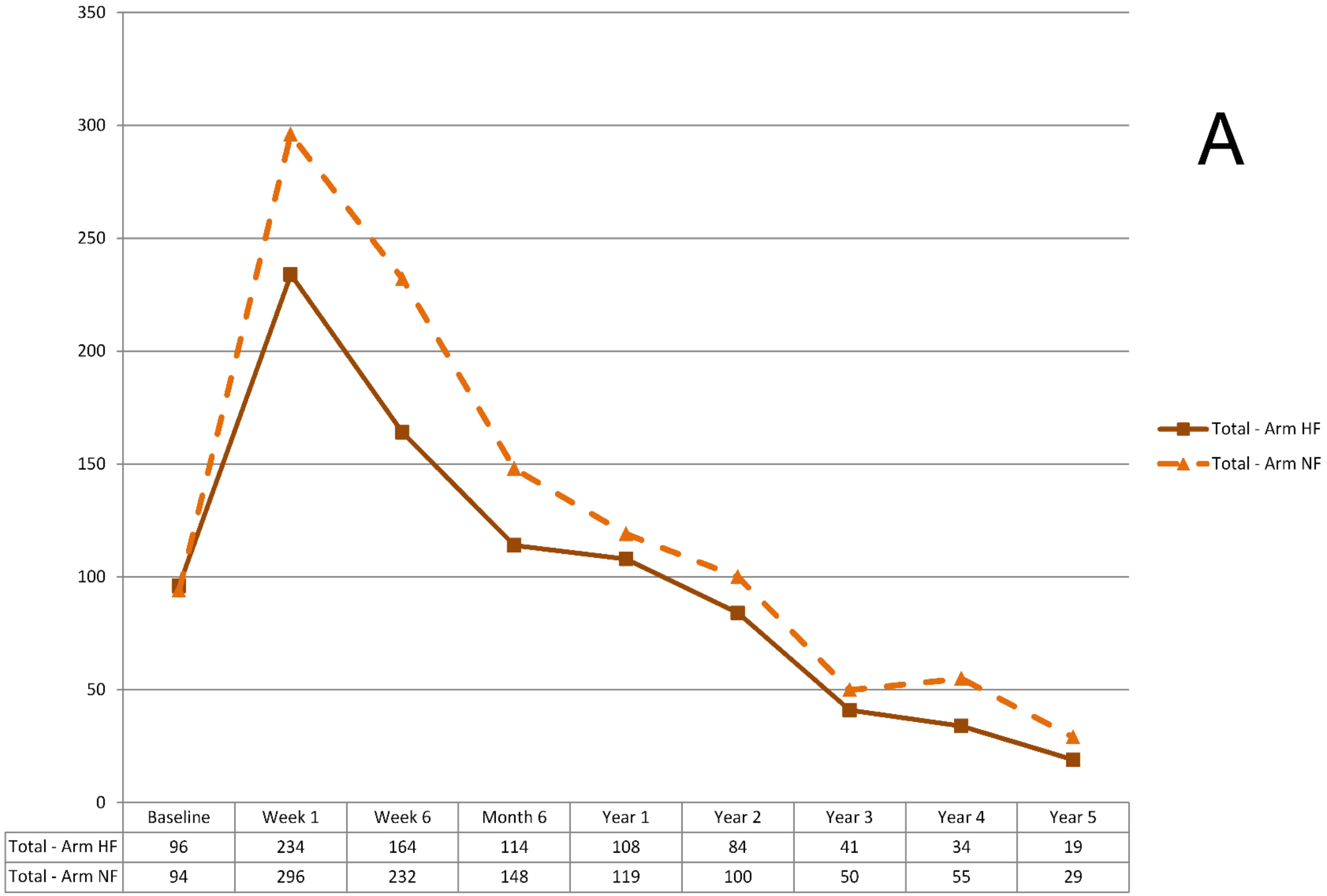

	Baseline	Week 1	Week 6	Month 6	Year 1	Year 2	Year 3	Year 4	Year 5
Total - Arm HF	96	234	164	114	108	84	41	34	19
Total - Arm NF	94	296	232	148	119	100	50	55	29

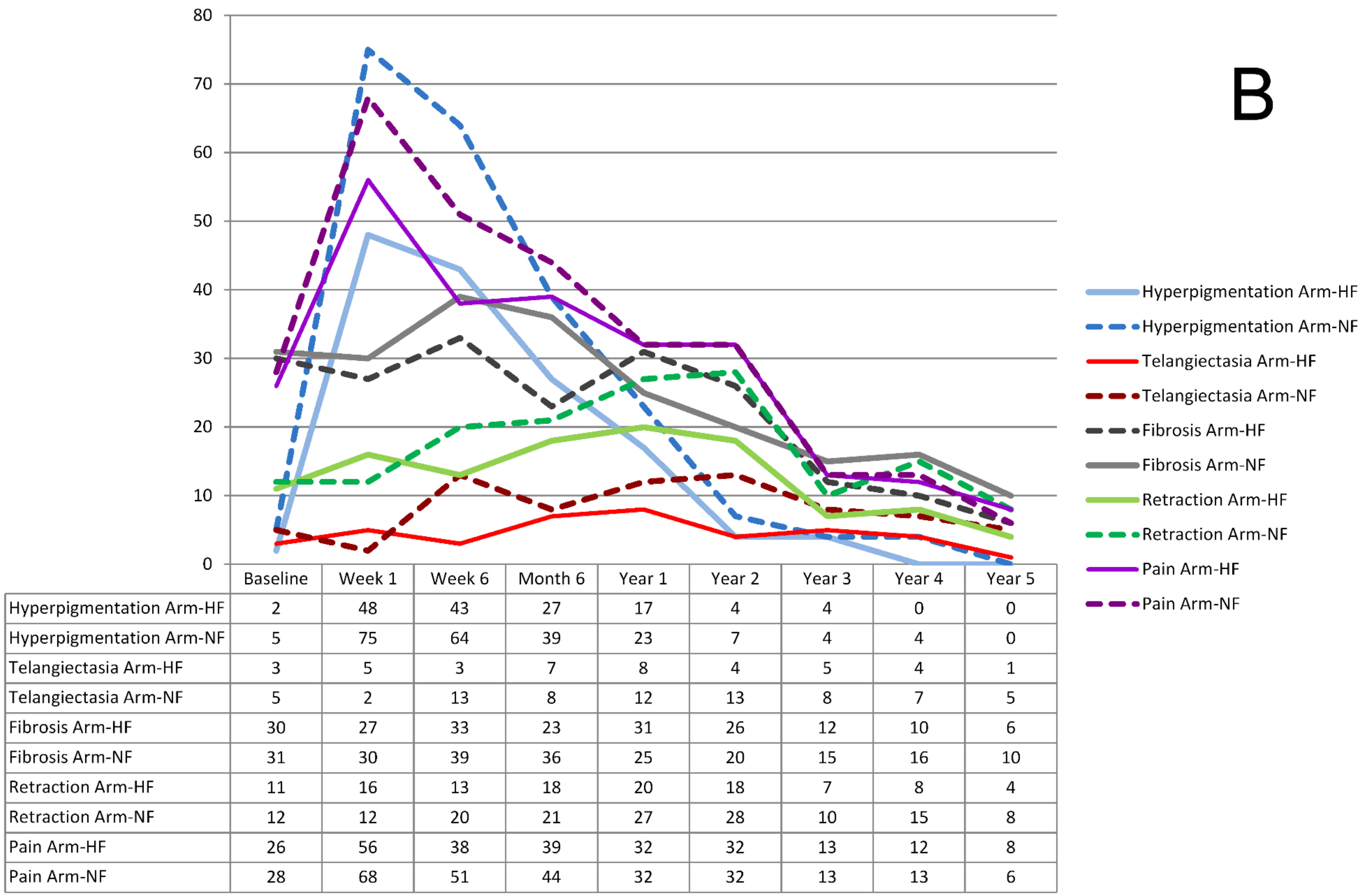

	Baseline	Week 1	Week 6	Month 6	Year 1	Year 2	Year 3	Year 4	Year 5
Hyperpigmentation Arm-HF	2	48	43	27	17	4	4	0	0
Hyperpigmentation Arm-NF	5	75	64	39	23	7	4	4	0
Telangiectasia Arm-HF	3	5	3	7	8	4	5	4	1
Telangiectasia Arm-NF	5	2	13	8	12	13	8	7	5
Fibrosis Arm-HF	30	27	33	23	31	26	12	10	6
Fibrosis Arm-NF	31	30	39	36	25	20	15	16	10
Retraction Arm-HF	11	16	13	18	20	18	7	8	4
Retraction Arm-NF	12	12	20	21	27	28	10	15	8
Pain Arm-HF	26	56	38	39	32	32	13	12	8
Pain Arm-NF	28	68	51	44	32	32	13	13	6

2. Absolute and relative numbers of events per treatment arm and time point, detailed by grading. A significant difference is highlighted when given.

Dermatitis		G0	GI	GII	G0 %	GI %	GII %		
Baseline	Arm HF	93	5	2	93.0	5.0	2.0	100	*Fisher Exact Test*
	Arm NF	111	6	0	94.9	5.1	0.0	117	*p=0.372*
Week 1	Arm HF	34	58	8	34.0	58.0	8.0	100	***χ^2-Test p=0.034***
	Arm NF	23	77	17	19.7	65.8	14.5	117	
Week 6	Arm HF	86	14	0	86.0	14.0	0.0	100	*χ^2-Test p=0.269*
	Arm NF	94	23	0	80.3	19.7	0.0	117	

Pneumonitis		G0	GI	GII	G0 %	GI %	GII %		
Baseline	Arm HF	99	1	0	*99.0*	*1.0*	*0.0*	100	*Fisher Exact Test*
	Arm NF	117	0	0	*100.0*	*0.0*	*0.0*	117	*p=0.461*
Week 1	Arm HF	99	0	1	*99.0*	*0.0*	*1.0*	100	*Fisher Exact Test*
	Arm NF	117	0	0	*100.0*	*0.0*	*0.0*	117	*p=0.461*
Week 6	Arm HF	96	0	4	*96.0*	*0.0*	*4.0*	100	***Fisher Exact***
	Arm NF	116	1	0	*99.1*	*0.9*	*0.0*	117	***Test p=0.044***

**Cough**		G0	GI	GII	G0 %	GI %	GII %		
Baseline	Arm HF	92	8	0	*92.0*	*8.0*	*0.0*	100	*χ²-Test p=0.249*
	Arm NF	112	5	0	*95.7*	*4.3*	*0.0*	117	
Week 1	Arm HF	91	8	1	*91.0*	*8.0*	*1.0*	100	*Fisher Exact Test*
	Arm NF	108	9	0	*92.3*	*7.7*	*0.0*	117	*p=0.794*
Week 6	Arm HF	89	11	0	*89.0*	*11.0*	*0.0*	100	*χ²-Test p=0.542*
	Arm NF	107	10	0	*91.5*	*8.5*	*0.0*	117	

**Dyspnea**		G0	GI	GII	GIII	G0 %	GI %	GII %	GIII %		
Baseline	Arm HF	92	7	0	1	*92.0*	*7.0*	*0.0*	*1.0*	100	*Fisher Exact Test*
	Arm NF	112	5	0	0	*95.7*	*4.3*	*0.0*	*0.0*	117	*p=0.305*
Week 1	Arm HF	94	6	0	0	*94.0*	*6.0*	*0.0*	*0.0*	100	*Fisher Exact Test*
	Arm NF	111	4	2	0	*94.9*	*3.4*	*1.7*	*0.0*	117	*p=0.407*
Week 6	Arm HF	85	13	2	0	*85.0*	*13.0*	*2.0*	*0.0*	100	*Fisher Exact Test*
	Arm NF	106	7	3	1	*90.6*	*6.0*	*2.6*	*0.9*	117	*p=0.197*

Hyperpigmentation		G0	GI	GII	G0 %	GI %	GII %	Patients	
**Baseline**	**Arm HF**	98	2	0	*98.0*	*2.0*	*0.0*	100	*Fisher Exact*
	**Arm NF**	112	5	0	*95.7*	*4.3*	*0.0*	117	*Test p=0.456*
**Week 1**	**Arm HF**	52	45	3	*52.0*	*45.0*	*3.0*	100	***χ²-Test***
	**Arm NF**	42	63	12	*35.9*	*53.8*	*10.3*	117	***p=0.017***
**Week 6**	**Arm HF**	57	39	4	*57.0*	*39.0*	*4.0*	100	*χ²-Test*
	**Arm NF**	53	52	12	*45.3*	*44.4*	*10.3*	117	*p=0.095*
**Month 6**	**Arm HF**	68	25	2	*71.6*	*26.3*	*2.1*	95	*Fisher Exact*
	**Arm NF**	73	33	6	*65.2*	*29.5*	*5.4*	112	*Test p=0.399*
**Year 1**	**Arm HF**	73	16	1	*81.1*	*17.8*	*1.1*	90	*Fisher Exact*
	**Arm NF**	86	20	3	*78.9*	*18.3*	*2.8*	109	*Test p=0.800*
**Year 2**	**Arm HF**	*78*	4	0	*95.1*	*4.9*	*0.0*	82	*Fisher Exact*
	**Arm NF**	98	7	0	*93.3*	*6.7*	*0.0*	105	*Test p=0.758*
**Year 3**	**Arm HF**	40	3	1	*90.9*	*6.8*	*2.3*	44	*Fisher Exact*
	**Arm NF**	*58*	4	0	*93.5*	*6.5*	*0.0*	62	*Test p=0.678*
**Year 4**	**Arm HF**	27	0	0	*100.0*	*0.0*	*0.0*	27	*Fisher Exact*
	**Arm NF**	43	4	0	*91.5*	*8.5*	*0.0*	47	*Test p=0.290*
**Year 5**	**Arm HF**	17	0	0	*100.0*	*0.0*	*0.0*	17	*Keine Statistik*
	**Arm NF**	26	0	0	*100.0*	*0.0*	*0.0*	26	
**Year 6**	**Arm HF**	11	*1*	0	*91.7*	*8.3*	*0.0*	12	*Fisher Exact*
	**Arm NF**	13	*0*	1	*92.9*	*0.0*	*7.1*	14	*Test p=0.720*
**> Year 7**	**Arm HF**	7	1	0	*87.5*	*12.5*	*0.0*	8	*Fisher Exact*
	**Arm NF**	*10*	0	0	*100.0*	*0.0*	*0.0*	10	*Test p=0.444*

Absolute incidence

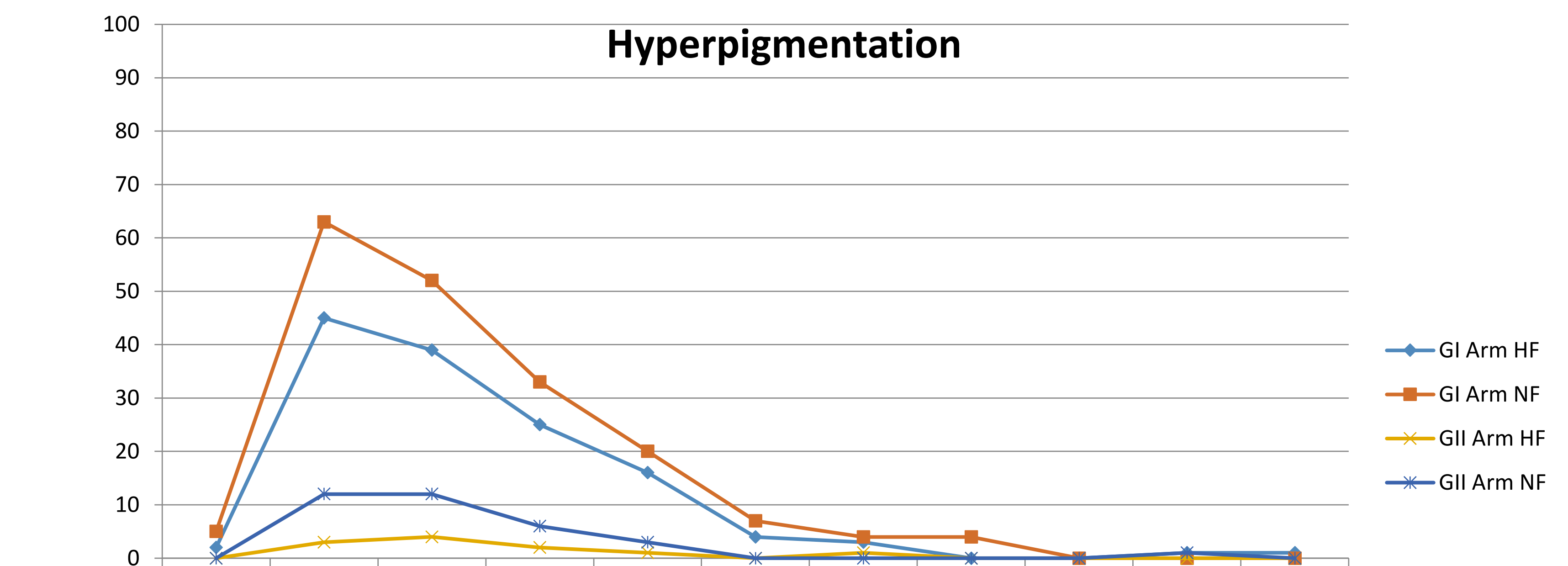

	Baseline	Week 1	Week 6	Month 6	Year 1	Year 2	Year 3	Year 4	Year 5	Year 6	> Year 7
GI Arm HF	2	45	39	25	16	4	3	0	0	1	1
GI Arm NF	5	63	52	33	20	7	4	4	0	0	0
GII Arm HF	0	3	4	2	1	0	1	0	0	0	0
GII Arm NF	0	12	12	6	3	0	0	0	0	1	0
Pat Arm HF	100	100	100	95	90	82	44	27	17	12	8
Pat Arm NF	117	117	117	112	109	105	62	47	26	14	10

Telangiectasia		G0	GI	G0 %	GI %		
**Baseline**	**Arm HF**	97	3	*97.0*	*3.0*	100	*Fisher Exact*
	**Arm NF**	112	5	*95.7*	*4.3*	117	*Test p=0.728*
**Week 1**	**Arm HF**	95	5	*95.0*	*5.0*	100	*Fisher Exact*
	**Arm NF**	115	2	*98.3*	*1.7*	117	*Test p=0.252*
**Week 6**	**Arm HF**	97	3	*97.0*	*3.0*	100	***χ^2-Test***
	**Arm NF**	104	13	*88.9*	*11.1*	117	***p=0.023***
**Month 6**	**Arm HF**	88	7	*92.6*	*7.4*	95	*χ^2-Test*
	**Arm NF**	105	8	*92.9*	*7.1*	113	*p=0.936*
**Year 1**	**Arm HF**	82	8	*91.1*	*8.9*	90	*χ^2-Test*
	**Arm NF**	97	12	*89.0*	*11.0*	109	*p=0.621*
**Year 2**	**Arm HF**	*78*	4	*95.1*	*4.9*	82	*χ^2-Test*
	**Arm NF**	92	13	*87.6*	*12.4*	105	*p=0.077*
**Year 3**	**Arm HF**	39	5	*88.6*	*11.4*	44	*χ^2-Test*
	**Arm NF**	*54*	8	*87.1*	*12.9*	62	*p=0.812*
**Year 4**	**Arm HF**	23	4	*85.2*	*14.8*	27	*Fisher Exact*
	**Arm NF**	40	7	*85.1*	*14.9*	47	*Test p=1.000*
**Year 5**	**Arm HF**	16	1	*94.1*	*5.9*	17	*Fisher Exact*
	**Arm NF**	21	5	*80.8*	*19.2*	26	*Test p=0.376*
**Year 6**	**Arm HF**	12	0	*100.0*	*0.0*	12	*Fisher Exact*
	**Arm NF**	10	4	*71.4*	*28.6*	14	*Test p=0.100*
**> Year 7**	**Arm HF**	7	1	*87.5*	*12.5*	8	*Fisher Exact*
	**Arm NF**	*6*	*4*	*60.0*	*40.0*	10	*Test p=0.314*

Absolute incidence

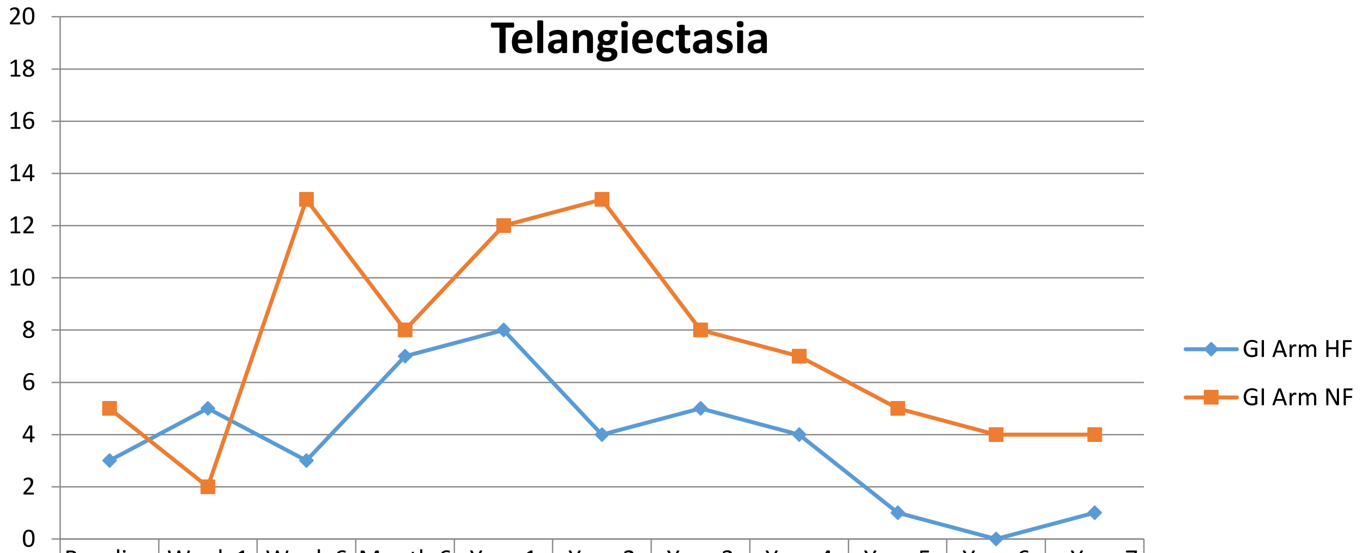

	Baseline	Week 1	Week 6	Month 6	Year 1	Year 2	Year 3	Year 4	Year 5	Year 6	> Year 7
GI Arm HF	3	5	3	7	8	4	5	4	1	0	1
GI Arm NF	5	2	13	8	12	13	8	7	5	4	4
Pat Arm HF	100	100	100	95	90	82	44	27	17	12	8
Pat Arm NF	117	117	117	113	109	105	62	47	26	14	10

Fibrosis		G0	GI	GII	GIII	G0 %	GI %	GII %	GIII %		
Baseline	**Arm HF**	70	23	7	0	*70.0*	*23.0*	*7.0*	*0.0*	100	*χ²-Test p=0.483*
	**Arm NF**	86	27	4	0	*73.5*	*23.1*	*3.4*	*0.0*	117	
Week 1	**Arm HF**	73	23	4	0	*73.0*	*23.0*	*4.0*	*0.0*	100	*Fisher Exact Test*
	**Arm NF**	87	26	4	0	*74.4*	*22.2*	*3.4*	*0.0*	117	*p=0.965*
Week 6	**Arm HF**	67	28	5	0	*67.0*	*28.0*	*5.0*	*0.0*	100	*χ²-Test p=0.959*
	**Arm NF**	78	34	5	0	*66.7*	*29.1*	*4.3*	*0.0*	117	
Month 6	**Arm HF**	72	18	5	0	*75.8*	*18.9*	*5.3*	*0.0*	95	*χ²-Test p=0.465*
	**Arm NF**	77	29	7	0	*68.1*	*25.7*	*6.2*	*0.0*	113	
Year 1	**Arm HF**	59	22	8	1	*65.6*	*24.4*	*8.9*	*1.1*	*90*	*Fisher Exact Test*
	**Arm NF**	84	19	6	0	*77.1*	*17.4*	*5.5*	*0.0*	109	*p=0.211*
Year 2	**Arm HF**	55	*18*	8	1	*67.1*	*22.0*	*9.8*	*1.2*	*82*	*Fisher Exact Test*
	**Arm NF**	85	16	3	1	*81.0*	*15.2*	*2.9*	*1.0*	*105*	*p=0.077*
Year 3	**Arm HF**	32	7	4	1	*72.7*	*15.9*	*9.1*	*2.3*	*44*	*Fisher Exact Test*
	**Arm NF**	*46*	*13*	3	0	*74.2*	*21.0*	*4.8*	*0.0*	62	*p=0.513*
Year 4	**Arm HF**	17	7	2	1	*63.0*	*25.9*	*7.4*	*3.7*	*27*	*Fisher Exact Test*
	**Arm NF**	32	13	0	*2*	*68.1*	*27.7*	*0.0*	*4.3*	*47*	*p=0.317*
Year 5	**Arm HF**	11	6	0	0	*64.7*	*35.3*	*0.0*	*0.0*	17	*χ²-Test p=0.834*
	**Arm NF**	16	10	0	0	*61.5*	*38.5*	*0.0*	*0.0*	26	
Year 6	**Arm HF**	4	5	2	1	*33.3*	*41.7*	*16.7*	*8.3*	*12*	*Fisher Exact Test*
	**Arm NF**	8	6	0	0	*57.1*	*42.9*	*0.0*	*0.0*	14	*p=0.220*
Year 7	**Arm HF**	1	2	4	1	*12.5*	*25.0*	*50.0*	*12.5*	*8*	***Fisher Exact Test***
	**Arm NF**	*6*	*4*	0	0	*60.0*	*40.0*	*0.0*	*0.0*	10	***p=0.016***

Absolute incidence

Fibrosis

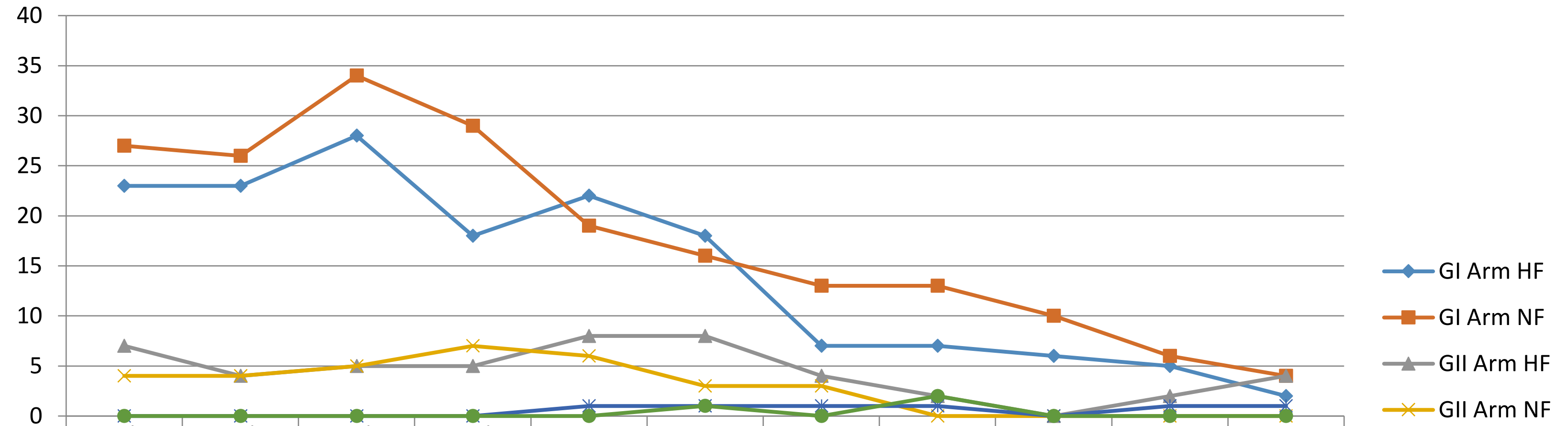

	Baseline	Week 1	Week 6	Month 6	Year 1	Year 2	Year 3	Year 4	Year 5	Year 6	Year 7
GI Arm HF	23	23	28	18	22	18	7	7	6	5	2
GI Arm NF	27	26	34	29	19	16	13	13	10	6	4
GII Arm HF	7	4	5	5	8	8	4	2	0	2	4
GII Arm NF	4	4	5	7	6	3	3	0	0	0	0
GIII Arm HF	0	0	0	0	1	1	1	1	0	1	1
GIII Arm NF	0	0	0	0	0	1	0	2	0	0	0
Pat Arm HF	100	100	100	95	90	82	44	27	17	12	8
Pat Arm NF	117	117	117	113	109	105	62	47	26	14	10

Retraction		G0	GI	G0 %	GI %		
Baseline	**Arm HF**	89	11	*89.0*	*11.0*	100	*χ²-Test*
	**Arm NF**	105	12	*89.7*	*10.3*	117	*p=0.859*
Week 1	**Arm HF**	84	16	*84.0*	*16.0*	100	*χ²-Test*
	**Arm NF**	105	12	*89.7*	*10.3*	117	*p=0.208*
Week 6	**Arm HF**	87	13	*87.0*	*13.0*	100	*χ²-Test*
	**Arm NF**	97	20	*82.9*	*17.1*	117	*p=0.403*
Month 6	**Arm HF**	*77*	18	*81.1*	*18.9*	95	*χ²-Test*
	**Arm NF**	92	21	*81.4*	*18.6*	113	*p=0.947*
Year 1	**Arm HF**	70	20	*77.8*	*22.2*	90	*χ²-Test*
	**Arm NF**	82	27	*75.2*	*24.8*	109	*p=0.674*
Year 2	**Arm HF**	64	18	*78.0*	*22.0*	82	*χ²-Test*
	**Arm NF**	77	28	*73.3*	*26.7*	105	*p=0.458*
Year 3	**Arm HF**	37	7	*84.1*	*15.9*	44	*χ²-Test*
	**Arm NF**	52	10	*83.9*	*16.1*	62	*p=0.976*
Year 4	**Arm HF**	19	8	*70.4*	*29.6*	27	*χ²-Test*
	**Arm NF**	32	15	*68.1*	*31.9*	47	*p=0.838*
Year 5	**Arm HF**	10	*7*	*58.8*	*41.2*	17	*χ²-Test*
	**Arm NF**	18	8	*69.2*	*30.8*	26	*p=0.484*

Absolute incidence

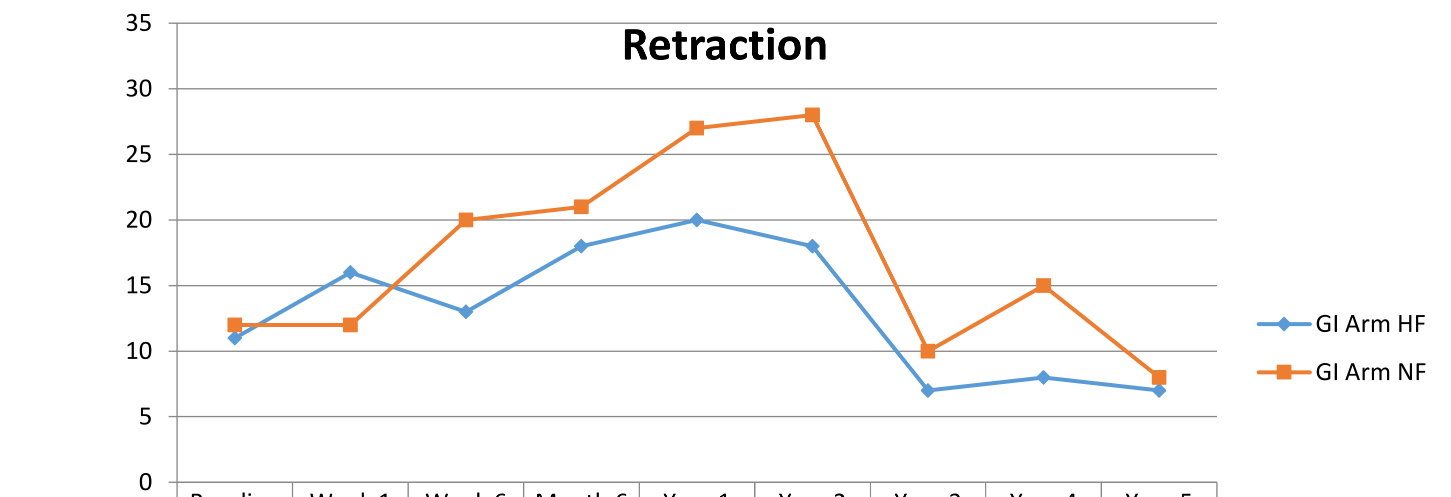

	Baseline	Week 1	Week 6	Month 6	Year 1	Year 2	Year 3	Year 4	Year 5
GI Arm HF	11	16	13	18	20	18	7	8	7
GI Arm NF	12	12	20	21	27	28	10	15	8
Pat Arm HF	100	100	100	95	90	82	44	27	17
Pat Arm NF	117	117	117	113	109	105	62	47	26

**Breast pain**		G0	GI	GII	GIII	G0 %	GI %	GII %	GIII %		
Baseline	**Arm HF**	74	19	7	0	*74.0*	*19.0*	*7.0*	*0.0*	100	*χ^2-Test p=0.841*
	**Arm NF**	89	22	6	0	*76.1*	*18.8*	*5.1*	*0.0*	117	
Week 1	**Arm HF**	44	35	20	1	*44.0*	*35.0*	*20.0*	*1.0*	100	*Fisher Exact Test*
	**Arm NF**	49	54	13	1	*41.9*	*46.2*	*11.1*	*0.9*	117	*p=0.159*
Week 6	**Arm HF**	62	23	12	3	*62.0*	*23.0*	*12.0*	*3.0*	100	*Fisher Exact Test*
	**Arm NF**	66	41	8	2	*56.4*	*35.0*	*6.8*	*1.7*	117	*p=0.176*
Month 6	**Arm HF**	56	25	14	0	*58.9*	*26.3*	*14.7*	*0.0*	95	*Fisher Exact Test*
	**Arm NF**	69	36	7	1	*61.1*	*31.9*	*6.2*	*0.9*	113	*p=0.130*
Year 1	**Arm HF**	58	23	8	1	*64.4*	*25.6*	*8.9*	*1.1*	90	*Fisher Exact Test*
	**Arm NF**	77	28	2	2	*70.6*	*25.7*	*1.8*	*1.8*	109	*p=0.136*
Year 2	**Arm HF**	51	20	*8*	3	*62.2*	*24.4*	*9.8*	*3.7*	82	*sher Exact Test p=0.2*[illegible]
	**Arm NF**	73	21	11	0	*69.5*	*20.0*	*10.5*	*0.0*	105	
Year 3	**Arm HF**	31	10	2	1	*70.5*	*22.7*	*4.5*	*2.3*	44	*Fisher Exact Test*
	**Arm NF**	49	12	1	0	*79.0*	*19.4*	*1.6*	*0.0*	62	*p=0.437*
Year 4	**Arm HF**	15	9	2	1	*55.6*	*33.3*	*7.4*	*3.7*	27	*Fisher Exact Test*
	**Arm NF**	34	11	2	0	*72.3*	*23.4*	*4.3*	*0.0*	47	*p=0.289*
Year 5	**Arm HF**	9	7	1	0	*52.9*	*41.2*	*5.9*	*0.0*	17	*Fisher Exact Test*
	**Arm NF**	20	4	2	0	*76.9*	*15.4*	*7.7*	*0.0*	26	*p=0.142*

Absolute incidence

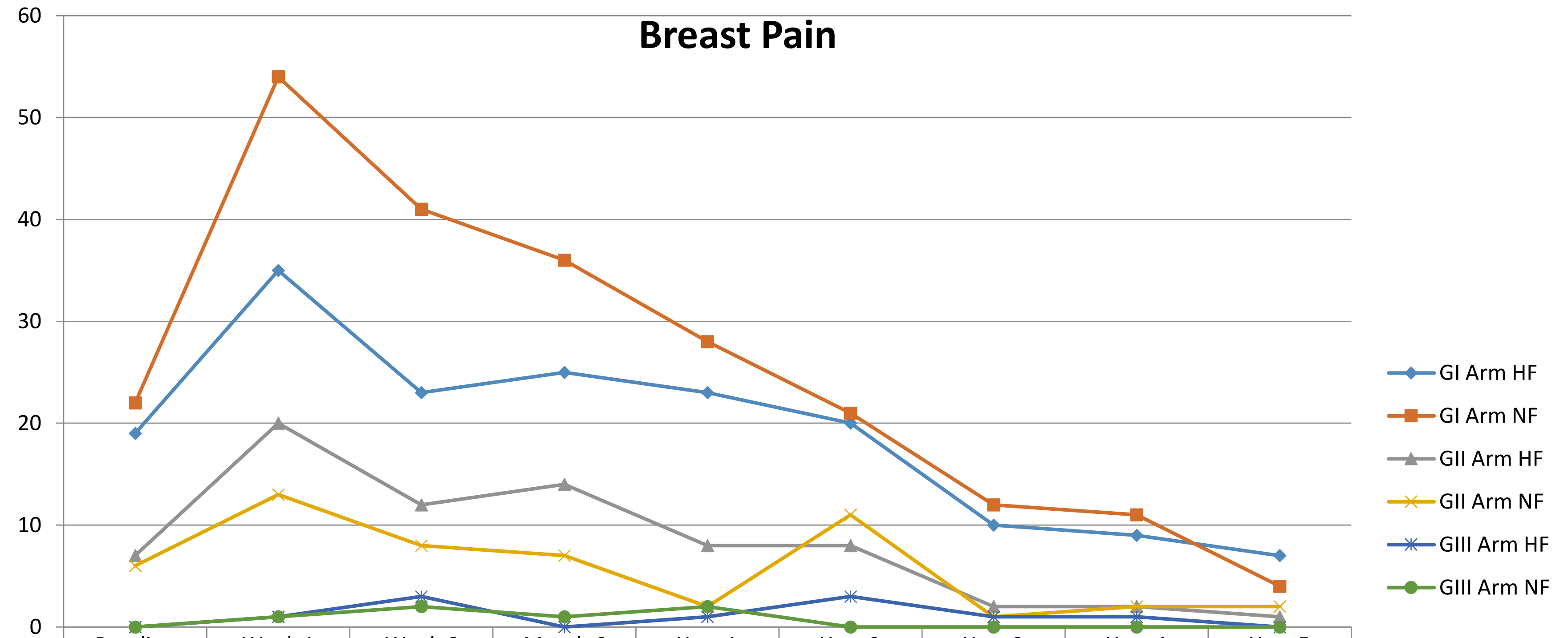

	Baseline	Week 1	Week 6	Month 6	Year 1	Year 2	Year 3	Year 4	Year 5
GI Arm HF	19	35	23	25	23	20	10	9	7
GI Arm NF	22	54	41	36	28	21	12	11	4
GII Arm HF	7	20	12	14	8	8	2	2	1
GII Arm NF	6	13	8	7	2	11	1	2	2
GIII Arm HF	0	1	3	0	1	3	1	1	0
GIII Arm NF	0	1	2	1	2	0	0	0	0
Pat Arm HF	100	100	100	95	90	82	44	27	17
Pat Arm NF	117	117	117	113	109	105	62	47	26
